# Mitophagy, a potential therapeutic target for stroke

**DOI:** 10.1186/s12929-018-0487-4

**Published:** 2018-11-30

**Authors:** Ruiqiao Guan, Wei Zou, Xiaohong Dai, Xueping Yu, Hao Liu, Qiuxin Chen, Wei Teng

**Affiliations:** 1 Heilongjiang University Of Chinese Medicine, Harbin, 150040 Heilongjiang province China; 2 First Affiliated Hospital of Heilongjiang University Of Chinese Medicine, Harbin, 150040 Heilongjiang province China; 3 Clinical Key Laboratory of Integrated Chinese and Western Medicine of Heilongjiang, University of Chinese Medicine, Beijing, 150040 China; 4 London South Bank University, London, SE1 6RD UK; 5 London Confucius Institute of Traditional Chinese Medicine, London, SE1 0AA UK; 6 Tonghe Hospital of Zhejiang Province, Ningbo, 315099 Zhejiang province China

**Keywords:** Mitochondria autophagy, Mitochondria, Stroke

## Abstract

Mitochondria autophagy, termed as mitophagy, is a mechanism of specific autophagic elimination of mitochondria. Mitophagy controls the quality and the number of mitochondria, eliminating dysfunctional or excessive mitochondria that can generate reactive oxygen species (ROS) and cause cell death. Mitochondria are centrally implicated in neuron and tissue injury after stroke, due to the function of supplying adenosine triphosphate (ATP) to the tissue, regulating oxidative metabolism during the pathologic process, and contribution to apoptotic cell death after stroke. As a catabolic mechanism, mitophagy links numbers of a complex network of mitochondria, and affects mitochondrial dynamic process, fusion and fission, reducing mitochondrial production of ROS, mediated by the mitochondrial permeability transition pore (MPTP). The precise nature of mitophagy’s involvement in stroke, and its underlying molecular mechanisms, have yet to be fully clarified. This review aims to provide a comprehensive overview of the integration of mitochondria with mitophagy, also to introduce and discuss recent advances in the understanding of the potential role, and possible signaling pathway, of mitophagy in the pathological processes of both hemorrhagic and ischemic stroke. The author also provides evidence to explain the dual role of mitophagy in stroke.

## Background

Stroke is associated with high mortality, and even those victims who survive are frequently left with severe neurological disorders, including cognitive, effective and sensorimotor dysfunction. Stroke is an acute cerebrovascular event, which may be classified as ischemic stroke or hemorrhagic stroke according to pathogeny. Ischemic stroke is caused by atherosclerotic plaque disruption with superimposing thrombosis, and spontaneous intracranial hemorrhage (ICH) is commonly caused by hypertension; other major causes include amyloid angiopathy, brain tumours, aneurysms, arteriovenous malformations and the use of anticoagulants [1–4]. For the treatment of ischemic stroke, Intravenous (IV) tissue plasminogen activator (IV tPA) is the only evidence-based therapeutic option for acute ischemic stroke patients and this has been the case since the 1990s [5], although intra-arterial thrombolysis (IAT) is a relatively safe and efficient short-term treatment for ischemic stroke which onset within 4.5 h [6]. In recent years endovascular procedures, including injecting thrombolytic agents into the thrombus, mechanically disrupting the clot by microwires and microcatheters, and percutaneous angioplasty has led to a tremendous development of stroke treatment [7]. In cases of cerebral hemorrhage, surgical decompression is a widely accepted, life-saving therapeutic method [8]. However, there are still limitations to the existing treatment, since the therapeutic benefit of IV tissue plasminogen activator (tPA) peaks within 90 min of symptom onset and lasts no longer than 4.5 h [9]. Furthermore, it may cause intracranial hemorrhage (ICH), which can worsen the neurologic impairment, or prove fatal. The effectiveness of endovascular revascularisation for stroke treatment has been evidenced by studies of rigorously selected patients who fulfill specific criteria [10].

Stroke research, including that of treatment targets, has evolved tremendously, but due to the benefits of enhancing the intrinsic ability of neurons, self-repair is currently the potential therapy most favored by many researchers. Among the multiple, intertwined mechanisms of endogenous defences against stroke injury, regulating mitochondrial bioenergetics is seems to be a promising line of enquiry. The event converging on mitochondria which induce cell death and tissue infarction after ischemic stroke is mainly the decreasing of ATP due to lack of nutrient and oxygen [11]. During the reperfusion phase, resupply of oxygen and nutrients would induce a series of cellular damage due to overloading of mitochondrial Ca2+, mitochondrial permeability transition pore (MPTP) opening and over production of reactive oxygen species (ROS) [12]. Whereas in the hemorrhagic stroke, mitochondrial dysfunction caused mitochondrial respiratory decrease but not ischemia is responsible for reduced oxygen metabolism in hematoma after ICH [13]. Of the ischemic stroke therapies, mitochondrial antioxidant targeting is identified to be the most promising one. However, it lack of successful clinical model, due to the currant antioxidants are having difficulty in penetrating into blood brain barrier [14]. Of the hemorrhagic stroke, pathway involving Keap1 (Kelch-like ECH-associated protein 1) and Nrf2(nuclear factor erythroid 2-related factor 2), a major endogenous antioxidant system is considered as a key therapeutic which can limiting the over production of ROS by mitochondria after ICH [15]. Though there is no consensus of the therapeutic after ICH targeting on mitochondria, this pathway may contributing to the development of antioxidant against neutralize oxidative stress.

Autophagy is defined as a regulated mechanism of degrading and recycling of cellular components participating in organelle turnover and protein quality control, which can prompt a response to stress conditions, including starvation, and pathological stress such as oxidative stress [16]. A constitutive role of autophagy is to prevent the accumulation of random molecular impairment in long-lived organelles, particularly mitochondria [17]. Mitochondria fulfil central roles in energy provision by oxidative phosphorylation, and its other crucial functions include energy metabolism, amino acids production, synthesis of lipids, and ion homeostasis [18]. However, mitochondria can also be death-promoting organelles, being the main source of excessive reactive oxygen species (ROS), and its ability to induce apoptosis by the release of pro-apoptotic factors such as cytochrome C, which may finally lead to disrupted ATP synthesis [19]. Mitochondrial autophagy acts as a mechanism of mitochondrial quality control; dysfunctional mitochondria can be recognised and removed by autophagy, selectively. During this process, mitochondria are recognised for selective autophagy by PINK1 and Parkin, mitophagy receptor Nix and Bnip3 and other related modulators [20]. Mitochondrial quality control, by autophagy or ubiquitin-proteasome system (UPS) machinery, has consequently become one of the most attractive targets for therapeutic intervention in neurodegenerative diseases, such as Parkinson ‘s and Alzheimer’s diseases, as well as for cardiovascular diseases including myocardial infarction [21]. However, exactly how mitophagy interacts with mitochondria and contributes to specific pathological stages in stroke has not been clarified. The threshold at which mitophagy undertakes a protective role in stroke by mitochondrial quality control also requires consideration.

This review aims to provide a synopsis of mitochondrial mechanisms integrated with mitophagy, and discusses our current understanding of how these processes are performed during stroke (both of ischemic and hemorrhagic). This paper will also speculate on the possibility that targeted manipulation of mitophagy may be exploited for the rational design of novel therapeutic interventions.

### Mitochondria contributes in stroke

Mitochondria are key regulators of cell fate. They can either promote cell survival, by producing ATP to motivate cellular activity, or partake in the process of apoptosis that leads to cell death. In this section, we provide a summary of mitochondrial mechanisms involved in ischemic stroke.

### ATP-dependence channels in stroke

Stroke, especially ischemic stroke, leads to the disturbance of oxygen and glucose supply, which results in impaired mitochondrial oxidative phosphorylation [22]. The process can be reflected by depletion of ATP, which may prompt a cascade of events. Loss of ATP after ischemic injury of brain cells disrupts the ionic gradients across the membranes, which are regulated by Na+/K+ ATPase [23]. These conditions, followed by increases in extracellular K+ and reversal of the amino acid transporters, trigger an increase in free cytosolic Ca2+, and increasing cytosolic free Ca2+ concentration may conversely overload the mitochondrial proton circuit, promote accumulation of extracellular glutamate, thus inducing excitotoxicity after stroke [24]. Deryagin et al. have reported that ischemic preconditioning (IPre) activated by diazoxide 24 h before ischemia decreased the focal area by 37%, and K + ATP channel blocker glibenclamide administration reversed the action of IPre. Additionally, blood NO metabolites were analysed in this research, indicating that haemoglobin-bound NO complexes in the R-conformation stored and carried NO to the tissues in ischemic stroke [25].

### Oxidative stress as determinant after stroke

Resupply of nutrients and oxygen during the reperfusion phase after stroke, re-activates mitochondrial aerobic respiration and leading to the production of ROS [26]. ROS generation would provoke the blood–brain barrier (BBB) breakdown and aggravate brain oedema [27], in terms of pathology it may lead to protein damage, lipid peroxidation and DNA destruction [28]. ROS generation overwhelm the antioxidant capacity of antioxidant enzymes, can also result in cell death during the stroke [29]. Superoxide dismutase (SOD), especially SOD2 (manganese SOD) catalysing the superoxide has been considered as a critical target for ischemia protection [30]. Research has demonstrated that MnSOD plays a crucial role in mitochondrial protection and against oxidative stress-induced cell death after ischemia/reperfusion, by detecting the mitochondrial superoxide anion radical production of Sod2−/+ mice with a hydroethidine (HEt) oxidation method [31]. In a recent study, activating biological antioxidant by Astaxanthin (ASX) in a middle cerebral artery occlusion (MCAO) rat model had a protective action against brain injuries. The results showed that the relevant mechanisms involved suppression of ROS, upregulating the expressing of SOD, and reduced expression of MDA(malondialdehyde,a high-level oxidative stress maker) [32]. Moreover, biological antioxidant activation may also inhibition of apoptosis by increasing Bcl-2 expression, and promote regeneration and survival of neurons by increasing the expression of GFAP, MAP-2, BDNF, and GAP-43 [33]. In this research, though the importance of maintaining the mitochondrial function was not directly stated, It was reflected by showing activate endogenous antioxidant by suppressing ROS may have protective effect against the cell death after stroke.

### Mitochondrial-dependent apoptosis induced by stroke

Apoptosis involving mitochondria is one of the cascade events that follows metabolic change during a stroke. The cytochrome c, and other mitochondrial proteins that release cytosol from the intermembrane, are central to the intrinsic pathway of apoptosis [34]. In this phase, cytochrome c interacts with the protein cofactor Apaf-1 and procaspase-9 to form apoptosome, Caspase-3 and other caspases having been activated to proteolysis caspase-dependent DNase, resulting in internucleosomal fragmentation of DNA [35]. In the extrinsic pathway, it is not necessary for executioner caspase to induce a cell apoptosis with the involvement of mitochondria [36]. However, caspase-independent forms of apoptosis are closely regulated by mediator AIF, a flavoprotein localised in the mitochondria, which can be released from the inner-membrane to trigger apoptosis after permeabilisation of the outer membrane [37]. The process can also lead to DNA degradation which involves other proteins including cyclophilin A, procaspase-9 and Endonuclease G [38]. Also, Bcl-2 members proteins have been implicated in mitochondrial apoptosis process. They regulate mitochondrial apoptosis by controlling the permeabilisation of the outer mitochondrial membrane by BAX and BAK [39], or direct interactions with the mitochondria [36]. Research has shown that ischemic brain injury could be reduced by taurine administration, thereby modulating the actions of Bcl-2 family proteins, as well as preserving the mitochondria function and inhibiting mitochondrial apoptosis by reduction of the cytosolic cytochrome C and maintenance of the Bcl-xL/Bax ratio via blocking the expression of calpain and caspase-3 [40]. PARP1 is one of the factors triggering the AIF release from the mitochondria and its translocation to the nucleus [41]. There was a noticeable study showed that treatment by *Withania somnifera* (WS) has effect on both of antiapoptotic and antioxidant properties in ischemia stroke model [42], the neuroprotective effect of WS was by attenuates the level of PARP1 and inhibit AIF translocation to blocking caspase-independent apoptosis pathway, and antioxidative effects was exhibited by modulation of HO-1(HO1 is an VEGF inducer) and Sema3A(inhibit axonal growth). Such studies have confirmed the vital role that mitochondria-dependent apoptosis plays in stroke.

## Mitophagy: A quality control mechanisms of mitochondrial

Mitophagy is a process which mitochondrial-derived vesicles engulf selected mitochondrial cargos and deliver them to lysosomes or peroxisomes for degradation. Quality control of mitochondrial by mitophagy is crucial to the mitochondrial content and metabolism homeostasis. Mitophagy fine tunes the mitochondrial biogenesis and homeostasis is playing a key role to the cellular and organismal physiology. The imbalance between them may leading to the mitochondrial accumulation, increased oxygen consumption, and excessive ROS generation, which eventually consequences as cellular degeneration and activation of cell death pathways [43]. The mechanism was investigated in the yeast cell. Under the condition of nitrogen starvation, in the wild-type cells, mitophagy was initiated to encounter nitrogen starvation by suppressing the mitochondrial reactive oxygen species (ROS) production and degrading the excess mitochondria to eliminate the proliferation during respiratory growth. In contrast, mitophagy-deficient ATG32- or ATG11- knock-out cells failed to degraded the excess mitochondria, thus leading to a vicious circle for more ROS to damage the mitochondria, which has finally caused mitochondrial DNA deletion and the so-called “petitemutant” phenotype [44]. Further, reported by a vitro study, hepatitis B virus (HBV) disrupts mitochondrial dynamics led to the balance of mitochondrial dynamics shift toward fission via triggering dynamin-related protein (Drp1) translocation, thereby induced mitophagy to attenuate the virus-induced apoptosis, thus promoted cell survival, and even possibly viral persistence [45]. These evidence suggested that aberrant mitochondrial and mitophagy may disturb apoptosis impending due to accrued mitochondrial injury. A homeostatic feedback loop that integrates both of mitochondrial biogenesis and mitophagy was studied by K Palikaras, E Lionaki1, and their colleagues, they innovatively found that DCT-1 is a crucial mediator of mitophagy and also a contributes to the longevity of the *Caenorhabditis elegans* (C.elegans) under stress conditions. Mitophagy deficiency has activated mitochondrial retrograde signaling through the SKN-1 transcription factor, which has coordination with both of mitochondrial biogenesis genes and mitophagy by upregulating DCT-1 expression. The study had illuminated the involvement of mitophagy in overproliferation of damaged mitochondria and cell function declination during the aging [46]. In mammals, mitophagy, the mitochondrial quality control system can be activated by a various of cellular events, which including reticulocytes maturation, aging, oxygen deprivation, a pathology stress which leading to mitochondrial dysfunction, and also reported to be activated in paternal mtDNA elimination [47]. Ulk1-dependent, and Atg5-independent alternative autophagy was proved to be the dominant process of clearing mitochondrial from reticulocytes during erythrocyte differentiation. Evidenced by showing fetal definitive reticulocytes from mice with Ulk1-deficient and Ulk1/Atg5 double-deficient failed to clear their mitochondria by autophagy, whereas mitophagy (autophagic structures with engulfed mitochondria) was occurred in the wild-type and Atg5-deficient mice [48]. Moreover, during the process of reticulocytes maturation, NIX (BNIP3L) is a mediator of developmentally regulated mitochondrial clearance via selective incorporation of mitochondria into autophagosomes. However, the induction of macroautophagy does not mediated by NIX [49].

Effectors including Parkin/PINK1 pathway, Bnip3, NIX, and FUNDC1 have been identified to be involved in the mitophagy of mammals since mitophagy was discovered about a decade year before [50]. Besides the classic mitophagy mediators, newly identified pro-apoptotic protein of BCL-2 family member BCL2-L-13(BCL-2-like protein13), the mammalian homolog of ATG32, was confirming as a novel receptor for mitophagy [51]. Like other classic mitophagy effector, BCL2-L-13 share a common feature in maintaining an LC-3-interacting region(LIR) that interacts with LC-3 [52]. Among the mitophagy effectors, PINK1/Parkin pathway shows a different mediation of mitophagy by ubiquitylation-related process. In addition, PARK2 (Parkin RBR E3 ubiquitin protein ligase) was excessively studied as mitophagy mediator in recent years. PARK2 mediates mitophagy via recruited by full-length PINK1 after mitochondrial depolarization., it binding to PINK1 by a ROS-dependent manner, which was supported by study showing that PARK2 recruitment induced mitophagy can be enhanced by increasing ROS in Sirt−/− cells [53]. PARK2-Beclin1 interaction is essential in PARK2 translocation to mitochondria and PARK2-dependent mitophagy. The interaction may depends on the Beclin1 absent induced CCCP inhibition and PINK1 overexpression [54]. Understanding these factors and their mechanism in mitophagy will lay new foundations of how mitochondrial activities orchestrate cell fate and hint some novel areas of research about how mitochondrial quality control by mitophagy are involved in some physiology and pathology conditions.

Further, some multifaceted cascades which may leading to dysfunctional mitochondrial clearance by mitophagy, including ROS production, mitochondrial permeability transition pore (MPTP) opening, loss of membrane potential, mitochondrial fission and fusion. The mechanism of several scenarios and events and their involvement in mitophagy during the stroke will be introduced with detail in the following section.

### Mitophagy implicates mitochondria biology in stroke

Mitochondria are dynamic organelles which dictate not only cellular bioenergetics, but also specific signaling cascades that are involved in critical cellular functions and survival. As a catabolic mechanism, mitophagy links elements of a complex network of mitochondria, including mitochondrial fusion and fission, oxidative stress and the mitochondrial permeability transition pore (MPTP).

### Mitophagy orchestrates ROS in stroke

Reactive oxygen species (ROS) comprises a group of highly reactive molecules, which is part of products from cellular metabolism under both physiological and stress conditions [55]. In regular conditions, ROS participate in a package of intracellular and cellular reactions, including the regulation of phosphatases, cellular growth, differentiation, proliferation, and apoptosis [56]. Mitochondria are the main intracellular sources of ROS. Excessive ROS may cause mitochondrial depolarization, leading to mitophagy, a mechanism that plays a critical role in clearing ROS by suppressing oxidative stress [57]. ROS-mediated mitophagy is a negative feedback mechanism, which means mitophagy is considered to be neuroprotective by eliminating ROS generation, whereas some researchers hold that ROS-regulated excessive or insufficient mitophagy (autophagy) results in cell death [58].

The role of ROS in mitophagy, especially in PINK1-Parkin-dependent mitophagy, remains elusive. It may be due to the effects of ROS scavengers on the recruitment of Parkin, but this is not clear. The controversy has been addressed by Xiao B, Goh JY et al., showing ROS scavengers unable to inhibit Parkin accumulation onto mitochondria, however decreasing ROS by lowering concentrated carbonyl cyanide m-chlorophenylhydrazone (CCCP) or using ROS scavengers can lead to Parking translocation being blocked successfully [59]. In the model of mitochondrial respiration disturbance triggered mitophagy, which was also induced by CCCP, Parkin, ubiquitin, and p62 were shown to prime damaged mitochondria for mitophagy. However, in contrast to the previous example, ROS was not found to have participated in priming the mitochondria by promoting Parkin translocation; Nix, however, does involve in this event [60].

In a pathological condition like stroke, ROS was identified as contributing to the brain injury by generating programmed cell death or injury to BBB [61]. After SAH injury, VDAC (mitochondrial docking sites that promote mitophagy) may orchestrate ROS by interacting with LC-3 [62]. VSAC1siRNA administration decreases VDAC1 and LC-3 and increases ROS accumulation, whereas rapamycin-activated mitophagy results in increased LC3 expression and decreased ROS in brain tissue. It can be concluded that VDAC-regulated mitophagy may have acted a protective effect by reducing ROS and cell death after SAH. Reducing ROS by up-regulated mitophagy with melatonin administration was reported to inhibit NLRP3 inflammasome activation in SAH rat model. At 24 h after SAH, melatonin treatment further up-regulated mitophagy-mediated proteins PINK and Parkin, reduced ROS content, attenuated morphological changes in the mitochondria thus to inhibit the NLRP3 inflammasome activation [63]. However, none of the studies described above applied a ROS inhibitor, to further elucidate the role played by ROS-induced oxidative stress during or after stroke injury. This evidence tells us that ROS is a crucial factor in neuroinflammation and cytotoxicity after stroke, and its generation can be regulated by mitophagy. This is corroborated by other studies, in which augmented mitophagy-reduced accumulation of ROS was demonstrated using an oxygen-glucose deprivation (OGD) vitro model, OGD-generated ROS elevation was suppressed by methylene blue treatment, and the suppression of ROS can be reversed by 3-MA [64] It should be noted also that excessive ROS that is mainly generated by NADPH oxidases (NOX), is a response to the BBB disruption after cerebral ischemia [65]. The studies reviewed above tell us that more attention should be paid to the detection and location of ROS in the focal area of stroke. Thus, means by which to reduce excessive ROS generation by mitophagy can be better characterised.

## Mitophagy and mitochondrial permeability transition pore

Studies have shown that mitochondrial permeability transition is involved in both apoptosis and necrosis cell death. The opening of the permeability transition pore results in loss of mitochondrial membrane potential, mitochondria depolarisation, swelling and the transition of substances between the mitochondrial matrix and cytoplasm [66]. The process of the MPTP opening has been shown to be a response to mitochondrial calcium concentrations [67]. Ca2+ overloading, production of oxidant chemicals (ROS and RNS), oxidation of glutathione and a decrease in mitochondrial membrane potential can all be the factors that promote mitochondrial damage and trigger MPT [68, 69]. The event that activates MPTP may also occur in the perifocal area and penumbra after stroke, thus blocking the MPTP to preserve the integrity and function of mitochondria may play a positive role in stroke salvage [70]. The hypothesis is supported by extensive research, though a study using a transient middle cerebral artery occlusion (tMAO) rat model has presented a novel result, that the neuroprotective effect of MeValCsA (a nonimmunosuppressive analogue of CsA) is equipotent to the MPT inhibitor CsA in protecting the brain tissue from damage during the reperfusion phase after tMCAO [71]. CypD is a component of calcium-induced PT, which is targeted by, and interacts with, CsA to inhibits the opening of the MPTP [72]. Similarly, absence of CypD decreased infarct size by reducing the susceptibility of cells to death in (MCAO) model, whereas CypD were less susceptible to the cell death induced by H2O2 than WT counterparts [73]. Similarly, the absence of CypD decreased infarct size by reducing the susceptibility of cells to death in an MCAO model, whereas CypD were less susceptible to the cell death induced by H2O2 than WT counterparts. During the reperfusion phase of ischemic stroke, inhibiting MPT with NIM811 within a post-ischemic interval has reduced infarct volume, which highlighted that regulating MPT to prevent the collapse of the mitochondrial membrane potential may have a neuroprotective effect in stroke [74]. These studies strongly support the essential role of MPTP in stroke.

Mitochondrial permeability transition (MPT) is among the fundamental events in the initiation of mitochondrial autophagy (mitophagy),however, cell-repairing autophagy only occurs when there is a small portion of mitochondria undergoing MPT. When there is large quantity of mitochondria involved, autophagy is relatively inadequate, and may develop into apoptosis [75]. Consistently, autophagy induced in SH-SY5Y cells by 3-nitropropionic acid (3NP) treatment has shown constant mitochondrial morphology changes which are due to the mitochondrial swelling by the formation of MPTPs. These effects could be inhibited by CsA treatment, but not with molecular fission machinery, since 3NP does not recruit Drp-1 to mitochondria. Research results have indicated that autophagy preceding the apoptosis was proved to be mediated by MPTP [76]. Interestingly, unlike MPTP involved in autophagy, Bnip3 (Bcl-2 nineteen-kilodalton interacting protein 3) overexpression induces mitochondria sequestered by autophagosomes in cardiac myocytes has not shown permeability transition, and the mitophagy was not reversed by CsA treatment [77]. However, cypD-induced autophagy was equipotent to which by Bnip3 in both of the wild-type and cypD-deficient mice, which indicated that Bnip3 could induce mitophagy, possibly independent of the mPTP. PINK1-regulating mitophagy may be a response to the controlling of MPTP opening, which has been considered a probable molecular mechanism of MPTP-mediated mitophagy [78]. However, what we can be sure of is that MPTP is involved with the selective removal of damaged mitochondria by autophagy [79]. Further research is needed to delineate the mechanisms of MPTP involved in mitophagy, and to establish a better understanding of stroke.

### Mitophagy and mitochondria dynamics modulation in stroke

Mitochondria dynamic and mitophagy has fundamental connections with cell survival and the pathobiology of stroke. The dynamic processes of mitochondria, namely fission and fusion, are essential to the mitochondrial functions. Current observations are drawn from studies presuming that fusion is propitious to cell survival whereas fission facilitates cell death [80]. Fusion is regulated by optic atrophy 1 (Opa1), mitofusin 1 (Mfn1), and mitofusin 2 (Mfn2) [81]. Cell viability compromised by fusion involved with maintaining homeostasis of matrix metabolites decreases content heterogeneity and mitochondrial permeabilisation [82, 83]. Fission is involved in the quality control of mitochondria, when unhealthy components are segregated from the healthy mitochondrial network by fission; marked mitochondria are tagged to undergo autophagy and degraded [84]. Fission can also lead to mitochondrial renewal, redistribution, and proliferation into synapses [85].

There may exist a steady state of fission and fusion processes, which is responsible for mitochondrial degradation by mitophagy. Dynamin-related protein 1(Drp1), a member of the dynamin family of GTPases, which is the master regulator of mitochondrial fission [86]. Mitophagy can be down-regulated by the loss of Drp-1-mediated fission under pathological stress. Inhibiting Drp-1 expression by Mdivi1 decreased mitophagy, but didn’t change the expression level of LC3B and p62 in the CA3 of the hippocampus, indicated that Drp-1-mediated mitophagy by specifically initiating mitophagy rather than increasing the overall autophagy [87].

Fission and fusion are also implicated in preventing or promoting programmed cell death [88]. Besides Mdivi1’s inhibition of mitophagy by decreasing Drp-1, it had no effect on caspase-3 and cyt-c expression in a pMAO model, further experiment observed there was a significant up-regulating release of caspase-9, but not caspase-8, and these results indicate that decreased mitophagy by restraining mitochondria fission has an effect on mitochondria-related apoptosis but not on the death receptor-mediated apoptosis [89]. In line with this notion, decreasing Drp-1 expression by carnosine treatment can attenuate the release of AIF and cytochrome C from mitochondria to the cytosol, and decreased mitophagy by inhibiting Parkin.

recruitment in the ischemia brain damage at same time [90]. These results imply that the inhibition of mitophagy by modulation of the mitochondria dynamic plays a neuroprotective role by finally reducing apoptosis in ischemic stroke. The “dual nature” of mitophagy is discussed elsewhere in this review.

There is now consensus among researchers that in PINK1/Parkin- mediated mitophagy the mitochondrial dynamics favor fission. In the brain neurons, PINK1 or Parkin overexpression pushes the mitochondrial fission/fusion dynamics toward fission, and PINK1 deficiency tips the balance towards more fusion and enhanced mitophagy [91]. This might be because physiology always tends to decline, and fission allows mitochondria to be more easily captured by autophagosomes. Also, excessive fission is induced by Drp-1 overexpression, and fusion inhibited by knocking down OPA1-suppressed PINK1 RNAi-induced mitochondrial morphological defect [92]. The mutant strains of Parkin or Pink1 can be partially complemented by restraining fusion proteins like Opa1 and Mitofusin [93]. Mdivi-1 (an inhibitor of Drp-1) regulation of PINK1 and Parkin changing in TBI was first studied by Q Wu, C Gao et al. [94], whereby inhibiting mitochondria fission by Mdivi-1 led to the decrease of PINK1 and Parkin expression compared to the vehicle group, indicated that inhibition of mitochondrial fission blocked mitophagy specifically. It is notable that Mdivi-1 treatment also decreased colocalization of LC3 puncta with TUNEL-positive signal, thus it may be concluded that inhibiting Drp-1 may also induce non-selective autophagy and activate anti-apoptosis pathway, thus to play a neuroprotective role after TBI. Indeed,in neurons Drp-1 and Parkin may not work interdependently, but synergistically, they are overlapping in mitochondria turnover. A Parkin-independence form has been shown [95], in which the loss of Drp-1 increased mitochondrial division and mitophagy reduction, which led to P62 (a mitophagy adaptor protein) and ubiquitinated proteins accumulating on mitophagy. Parkin absence, in contrast, caused no Purkinje neuron degeneration, which could imply that Drp1 and Parkin work along different pathways. Given the findings above, more comprehensive study of the underlying mechanism of mitochondria dynamics and mitophagy may provoke more novel thinking in terms of stroke prevention and treatment.

### Mitophagy in stroke

**Fig. 1:**
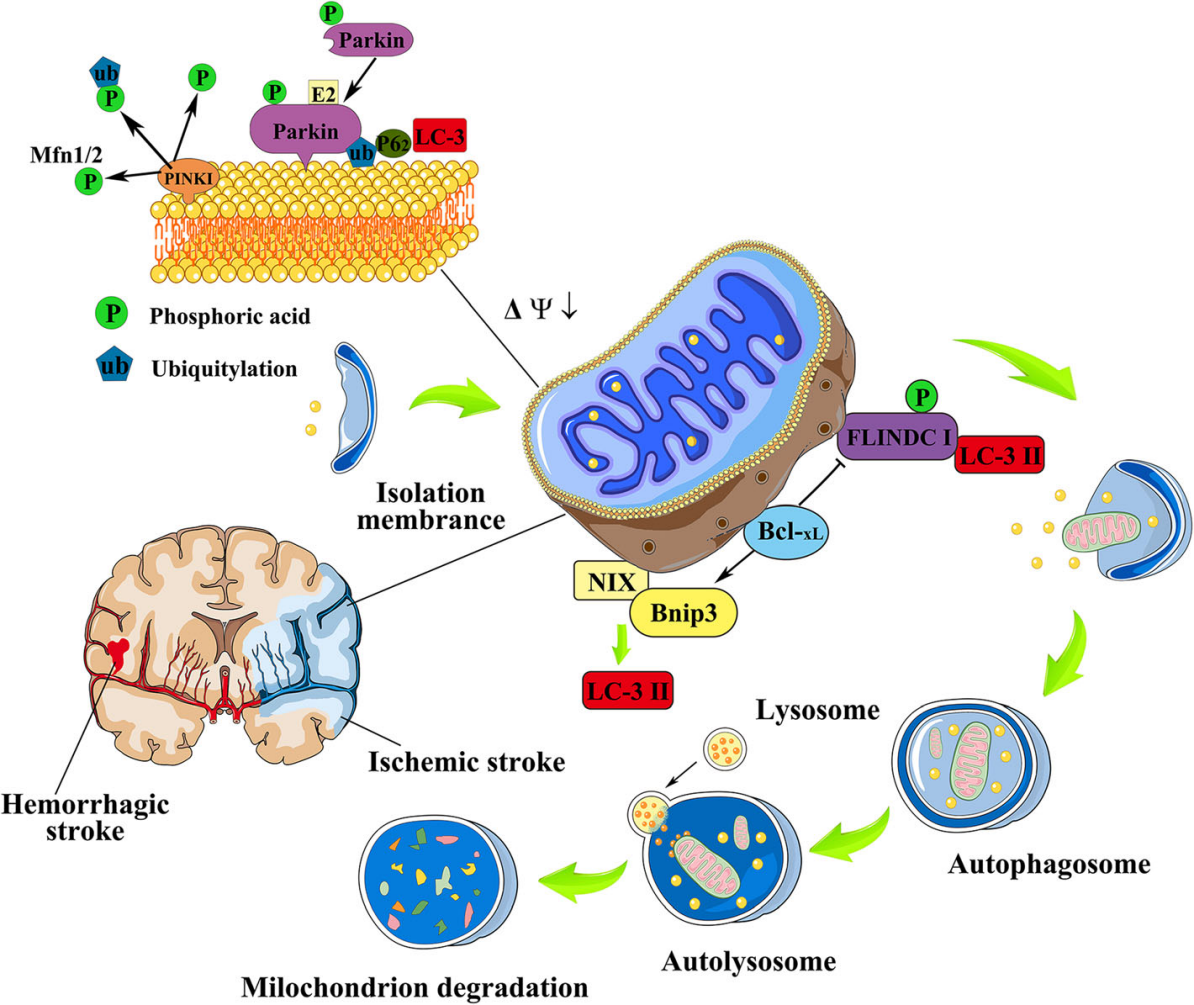
The molecular mechanism behind mitophagy after stroke. PINK, Parkin, NIX, BNIP3, and the newly-found FUNDC1 are mitophagy receptors in mammalian cells. Mitophagy regulated by PINK1-Parkin-mediated pathway is a multi-step process. Briefly, PINK1 is accumulated on the outer membrane of dysfunctional mitochondria, subsequently to recruit parkin by activating parkin’s cytosolic E3 ubiquitin ligase, then proteins on the outer mitochondrial membrane such as VDAC1 and Mfn1/2 are ubiquitinated by parkin to induce mitophagy. Adapter proteins including p62 are accumulated in the outer mitochondrial membrane after the ubiquitination, leading to ubiquitylated cargo recruited into autophagosomes by binding to LC3. BNIP3 and NIX are related multi-functional outer mitochondrial membrane proteins. Bnip3 and NIX activated mitophagy by binding to Bcl-2 family proteins (including BCL2 and BCL-xL), and also by repressing mTOR or regulating the production of ROS. The molecular mechanism of FUNDC1-mediated mitophagy has not been reported in the pathologies of stroke so far. FUNDC1 phosphorylated at serine 17 under hypoxia stress, thereby to interacting with LC3 and promoting mitophagy. Bcl-xL can inhibit LC-3II conversion, thereby suppressing FUNDC1-mediated mitophagy. In response to the stress of stroke (both of ischemic and hemorrhagic stroke), mitophagy is activated as a stress adaptation to removing dysfunctional mitochondria. Elongated mitochondria were divided into pieces, of which the process is preceded by mitochondrial fission, then autophagosomes, double-membrane vesicles are formed, and sequester targeted cell constituents and mitochondria. The mature autophagosomes then fuse with a lysosome to form the autophagolysosomes, where the mitochondria are subsequently degraded

Considerable advances have been made to elucidate the molecular mechanism behind mitophagy, as Fig. 1 illustrates. Hitherto PINK, Parkin, NIX, BNIP3, and newly-found FUNDC1 have been identified as mitophagy receptors in mammalian cells. In this section, we introduce signalling events regulating these mitophagy receptors and their physiological significance in stroke.

### BNIP3-regulated mitophagy in stroke

BNIP3 and BNIP3-like (BNIP3L) protein (NIX) are the homologous proteins of the Bcl-2 family; they share many key features such as a putative BH3 domain, mitochondrial outer membrane locations, and all induce apoptosis by C-terminal transmembrane domain [96]. The BNIP3 and NIX involved in mitophagy (autophagy) may be mechanistically independent, but are functionally related, as Fig. 1 shows. Since the putative BH3 domain of BNIP3 is different from other members of the Bcl-2 family, this BH-3 only domain confers proapoptotic activity [97]; whereas the C-terminal transmembrane domain of Bnip3 is essential for mitochondrial localisation and plays a role in the interaction of BNIP3 with BCL2 or BCL-xL [98].

Studies of Bnip3 in brain was mainly focused on apoptotic and necrotic cell death which is caused by a complex of post-ischemic events. Bnip3 overexpression can be induced by hypoxiainducible factor (HIF) 1 during the hypoxia condition in cerebral ischemia. A study had indicated an altered permeability of the nuclear membrane after cerebral ischemia which may initiate abnormal Bnip3 expression, thereby leading to the cell death [99]. Since BNIP3 labeling in CA3 of hippocampus lasted only for 1 day and was not appeared in nucleolus, but it lasted for 2 days and induced neuron damage in CA1 [100]. Similarly, Bnip3 overexpression may also acting as a target gene which activated by HIF-1α to exert a apoptotic effects in focal cerebral ischemia rat model [101]. The apoptosis cell death regulated by Bnip3 was studied in cortical neurons, Bnip3 expression followed by translocation to the mitochondrial outer membrane which leading to mitochondrial dysfunction, mitochondrial release of cytochrome c to execute a caspase-dependent cell death [102]. This apoptotic process was also found in mitochondrial dysfunction caused by Aβ-induced oxidative stress, which may enhanced by Bnip3 expression [103].

Bnip3 and NIX proteins integrate apoptosis and mitophagy signalling at different signalling domains [104], thus crosstalk between mitophagy and apoptosis may affect neuron cell death during stroke. Research in physiology has shown that Bnip3 has a dual regulation of Bcl-2 family proteins, which function as inhibiting pro-apoptosis protein Bcl-2, activating Bax and LC3 interacting region-mediated mitophagy, thereby suppressing apoptosis [105, 106]. Mitophagy response to hypoxia was first studied at organism level using the Spalax ehrenbergi (a species that can survive longer than other Rattus types under low oxygen conditions) as natural hypoxic tolerance model [107]. BNIP3 expression was reduced more significantly in Rattus than in S. Galili, indicating hypoxia tolerance is mediated by Bnip3-regulated mitophagy. Although the study did not involve the Bnip3 expression in brain cells, it suggests that improving the tolerance of hypoxia by regulating Bnip3 expression to reduce the ROS generalisation or DNA damage may also prove beneficial to organisms adapted to hypoxia stress after stroke.

The contribution of NIX in a cerebral ischemia-reperfusion (I-R) model was studied by Y Yuan, Y Zheng et al. [108], and the result implied that NIX would be a potential therapeutic target via regulating mitophagy in ischemic stroke. NIX knockout mice reduced mitophagy and aggravated cerebral I-R injury, overexpression of NIX compensated the injury and suppressed apoptosis by reducing CASP3 activation in the focal tissue after MCAO, which indicates the neuroprotective role of NIX. Interestingly, the Bnip3 was found to be decreased during reperfusion after ischemia. Though reinforced mitophagy was usually considered to improve neuronal survival,the stroke studies so far generally suggest that excessive mitophagy may play a negative roll in stroke. Therefore, like autophagy, mitophagy can be seen as a double-edged sword, the details of which will be introduced in the following section.

Bnip3 and NIX share 56% sequence homology, and this structural similarity might explain why they have a similar function in regulating mitophagy [109], and are both considered bona fide mitophagy receptors. A very insightful study has demonstrated the interaction between Bnip3 and NIX in stroke, with Bnip3 gene-silencing leading to a decrease of mitophagy and a neuroprotective effect in response to both of the vivo and vitro stroke models. NIX was also activated, in a similar trend [110]. Notably, NIX expression was higher in Bnip3 KO tissues compared with wild-type tissues at each time point after ischemia/hypoxia, which maintained mitophagy at a stable level. The results indicated that NIX might express to compensate for the absence of Bnip3 gene, but not entirely replace it. Further, mitophagy inhibition by knockout Bnip3 was independent of the general autophagy; in contrast, the autophagy was enhanced by upregulation of autophagic markers such as Beclin 1, LAMP2 and LC3II/I ratio. This perhaps suggest that NIX might function to maintain the mitophagy on a physiological level, but how Bnip3 functions has not yet been determined and must be further investigated. The roles of NIX and Bnip3 in mitophagy during stroke have challenged their conceptions of them as pro-apoptotic proteins; this also raises the question of how NIX and Bnip3 can be both neuroprotective and detrimental in stroke. Therefore, Bnip3 is a promising target for the control of cell survival or death by regulating mitophagy after stroke.

### PINK-Parkin-mediated mitophagy in stroke

Though mitochondria-specific autophagy was discovered in the 1960s, the specialised molecules that tag the dysfunctional mitochondria and submit them for autophagic degradation were not known until Parkin, an E3 ubiquitin ligase, was found by Richard Youle and his colleagues nearly 50 years later [111]. PINK1(phosphatase and tensin homolog (PTEN)-induced putative protein kinase 1) is a serine/threonine-protein kinase that contains an N-terminal mitochondrial targeting sequence (MTS), and upstream of Parkin in the same genetic pathway [112, 113].

Although it is not clear whether excessive mitophagy is protective or destructive, PINK1–Parkin-dependent mitochondrial maintenance has been considered to be dependent on mitophagy (see Fig. 1). A landmark study showed that PINK1 recruited to mitochondria with Parkin was associated with LC3, because the overexpression of both PINK1 and Parkin was colocalized, mainly with LC3-positive vesicles and partially with perinuclear aggregated mitochondria, which were expected to colocalize with aggregated Parkin [114]. However, PINK1 is required, but may not be indispensable for Parkin recruitment, which, the absence of PINK1 dose not inhibit Parkin translocation to mitochondria permanently [115, 116], but only delay the redistribution of Parkin to mitochondria [117]. Further, VDAC1 overexpression has no effect on PINK1 levels but can induce Parkin translocation [118]. Studies to investigate the mechanism of how PINK1 activates and recruits Parkin to mitochondria has shown that, though PINK1 directly phosphorylates ubiquitin, other substrates of PINK1 activate Parkin. For example, Parkin translocation to mitochondria may be independent of S65, as the mutation of all serine and threonine residues conserved between Drosophila and human did not totally inhibit Parkin translocation [119]. Further, decreased PINK1 expression by RNAi may inhibit ATP synthesis and also reduced autophagic flux, which can be restored by Parkin overexpression [120].

Absence of PINK1 in human dopaminergic neuroblastoma SH-SY5Y cells and differentiated neurons was proved to increase oxidative stress and mitochondrial impairment [121]. The PINK1–Parkin pathway also connected mitochondrial dynamic and mitophagy. In CCCP-induced mitophagy, the ubiquitination of fusion-related proteins mitofusins 1 (MFN-1) and 2 (MFN-2) both depended on PINK1 and Parkin [120]. In rat hippocampal neurons, silencing PINK1 led the mitochondrial fission/fusion dynamics to trend towards fusion, and inhibiting and overexpressing the Parkin gene in hippocampal neurons may increase and decrease the excitatory glutamatergic synapses respectively [91]. These results indicate that the pro-fission effect of the PINK1/ Parkin pathway might be one of the potential mechanisms for mitophagy initiation involved with mitochondrial maintenance. However, whether PINK1/Parkin is involved in the mitochondrial dynamic directly regulating the mitophagy neurons needs to be further investigated. In a study of cerebral ischemic damage in rats [122], increased p-Drp1 was in direct proportion to Parkin expression, after ischemic injury. The p-Drp1 and Parkin level was attenuated by carnosine treatment; furthermore, cytochrome C and apoptosis-inducing factor (AIF) were decreased in brain mitochondria. Taken in combination with previous research, it can be concluded Parkin may be involved in both mitophagy regulation and mitochondrial fragmentation in ischemic stroke model.

ROS has been presumed as a trigger for Parkin/PINK1-dependent mitophagy. As PINK1-dependent Parkin translocation was showed only to be effective without antioxidants in mouse cortical neurons, and in the absence of DJ-1, a ROS regulator, may lead to ROS accumulation-induced Parkin recruitment and increased mitophagy [123]. In a study of mouse brain, in the mitophagy induced by cadmium, decreasing ROS by NAC or acetyl-L-carnitine (ALC) (by reduction of MMP), suppressed the Parkin accumulating to mitochondria and decreased PINK1 level at same time; however, inhibiting cadmium-induced mitophagy by Cyclosporine A (CsA) has been found to block the PINK1/Parkin pathway but had no effect on the level of ROS [124] . This result confirmed that ROS functions on the upstream of the PINK1/Parkin pathway, to regulate mitophagy.

It is known that proteins regulating apoptosis act as mediating factors of mitophagy. Bcl-2 family proteins may be implicated in various aspects of the mechanism maintaining mitochondrial homeostasis, which include Parkin/PINK1-dependent mitophagy [125]. Parkin translocation to depolarised mitochondria-induced mitophagy has been shown to be inhibited by pro-survival Bcl-2 proteins (including Bcl-xL and Mcl-1), and enhanced by BH3-only proteins (Bad, Bim, and Noxa) [126]. The contradiction between PINK1-mediated mitophagy and apoptosis was further illustrated by a study of mild traumatic brain injury (mTBI). The neuroprotective role of rapamycin treatment was achieved by enhancing mitophagy via up-regulating PINK1 and downregulating apoptosis factors caspase-3 and cyt-c [127].

Recent research has elucidated the underlying mechanisms of PINK/Parkin-mediated mitophagy at the reperfusion phase after cerebral ischemia [128]. Peroxynitrite-(ONOO−, a typical reactive nitrogen species) induced Drp-1 recruitment to trigger the PINK1/Parkin-mediated mitophagy was studied for the first time; ONOO− was obviously increased after at reperfusion phase in vivo model, meanwhile, Drp-1 recruitment to mitochondrial and PINK1/Parkin-mediated mitophagy was initiated, combined with decreased mitochondria cytochrome c expression, to decrease the infarct size. The question of whether mitophagy is a neuro-protective mechanism is still under debate, and we will discuss whether mitophagy promotes or inhibits apoptosis in stroke in the next section.

Investigation of the role of the PINK1/Parkin pathway in mammalian neurons is necessary, since mitophagy disturbance involved with the PINK1/Parkin pathway might be a prerequisite of stroke therapeutic research.

## FUNDC1-mediated mitophagy

FUNDC1(Fun14 domain-containing protein 1) is a newly-discovered mitophagy receptor, which regulates the programmed elimination of mitochondria by directly binding to LC3 under hypoxic conditions [129]. Figure 1 illustrates. Under conditions of hypoxia, ULK1 (a Ser/Thr kinases required for early autophagosome formation) is increased and accumulated to impaired mitochondria, followed by phosphorylating FUNDC1 at serine 17, thereby enhancing the interaction between FUNDC1 and LC3 to promote mitophagy [130]. FUNDC1-regulated mitophagy is independent of the mitophagy induced by NIX or Bnip3, for NIX showed less strong binding with LC3II, and both NIX and Bnip3 were found to be increased when the expression of FUNDC1 was attenuated during hypoxia-induced mitophagy [131]. Also, FUNDC1 was found to integrate mitochondrial fission and the subsequent mitophagy at the ER-mitochondrial contact site (MAM, mitochondria-associated membrane) [132]. This is corroborated by a recent study, which has reported that, in mammalian cells in hypoxia conditions, FUNDC1/calnexin association attenuates, and the exposed cytosolic loop of FUNDC1 interacts with Drp-1 instead during mitophagy. Conversely, silencing the FUNDC1 resulted in mitochondrial elongation and mitophagy inhibition [133].

In the pathological research, FUNDC1 mediated mitophagy has been extensively reported in cardiac ischemic injury. In cardiac ischemia-reperfusion injury (IRI), FUNDC1 mediated mitophagy was induced to degrade mitochondria selectively, and to inhibit apoptosis during the ischemia phase; however, after reperfusion, Ripk3 expression was increased to elevated the mitochondrial apoptosis by inhibiting FUNDC1-modified mitophagy, resulting in the amplified injury of cardiac myocytes and microvascular reperfusion [134]. The protective role of FUNDC1-mediated mitophagy in hypoxic cardiomyocytes was further proven by very recent research [135], whereby PEDF treatment exerted a protective effect by promoting FUNDC1-mediated mitophagy via increasing PKC-ɑ (protein kinase Cɑ). This study has also added weight to the view that FUNDC1-regulated mitophagy is independent of that which is induced by NIX or Bnip3, for the PEDF specific increased level of FUNDC1. However, some contradictory reports have been published, for example, blocking FUNDC1-required mitophagy by melatonin via activating PPARγ can reduce the platelet activation, thus elevating the cardiac I/R injury, which may have inspired the view that PPARγ/FUNDC1/ mitophagy pathway might be a promising future therapeutic strategy in some other mitochondrial-involving diseases [136]. Mechanisms linking FUNDC1 to mitophagy have emerged as a novel research direction for various kinds of disease [137], but the molecular mechanism of FUNDC1-mediated mitophagy in the pathologies of stroke has not been reported so far.

### Mitophagy: A double-edged sword in stroke

As all the evidence above shows, there is no unified theory of whether mitophagy plays a pro-survival or pro-death role in stroke. Like macroautophagy, mitophagy undergoes extensive crosstalk, and even shares some common regulators with apoptosis signalling, and this crosstalk between the mitophagy and apoptosis might be one of the reasons behind the dual role of mitophagy in pathology conditions of human disease, such as stroke. BH3-only proteins, like Bnip3 (also NIX), integrate autophagy, mitophagy and apoptosis pathways [104], Bnip3 initiating mitophagy by the interaction of its LC3-interacting region (LIR) with Atg8 proteins. Upregulating mitophagy by enhancing Bnip3–Atg8 interactions may act against apoptosis by lowering cytochrome c release capacity [105], and (MOMP) BH3-only proteins directly binding to and activating Bax and Bak may trigger mitochondrial outer membrane permeabilisation (MOMP)-induced canonical apoptosis [138]. However, during mitophagy, MOMP execution takes much less time than mitophagy [139, 140], yet after the MOMP, autophagy can in turn be limited by the apoptotic [141], thus there is complexity and uncertainty in the interaction between mitophagy and apoptosis. As we have seen, above [106], both pre-active mitophagy by delaying MOMP initiation with tBid activation or increasing mitochondrial heterogeneity in Bax/Bcl2 levels can suppress the cytochrome c release in a subpopulation of mitochondria. Moreover, it has also been suggested that the extent of mitophagy may depend upon the level of autophagic vesicles (AV) within the cell and even the necessity amount of AV which maximum to the mitochondria [106, 142]. However, other results showed mitophagy was suppressed by inhibiting apoptosis, since Parkin/PINK1-dependent mitophagy was suppressed by anti-apoptosis Bcl-2 family protein (Bcl-xL and Mcl-1) and was enhanced by pro-apoptosis BH3-only proteins [126, 143]. Bcl-2 family proteins are implicated in many processes linked to the mitochondrial function and homeostasis [144] and may act as a critical point of the balance between the apoptosis and mitophagy. Thus, the characteristic of the Bcl-2 family proteins global regulating mitochondrial network might be one of the mechanisms underlying the duality of mitophagy.

**Fig. 2:**
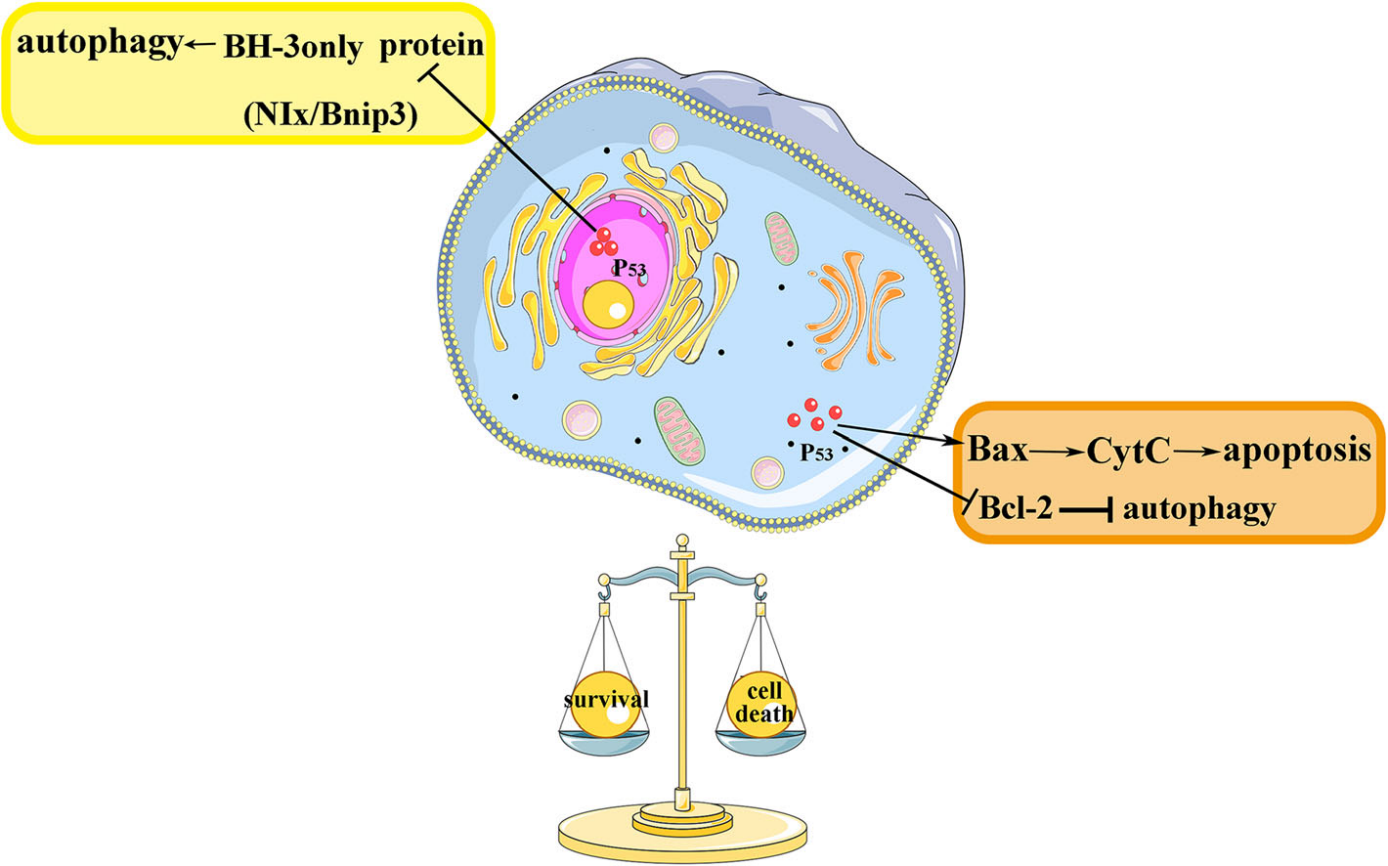
P53 and the dual role of mitophagy. P53 has overlapping alterations with Bcl-2 family protein. P53-involved intrinsic pathway of apoptosis, target Bcl-2 proteins and genes such as Bax, and subsequently induce mitochondrial membrane disruption and cytochrome c release. In autophagy regulation, nuclear P53 would induce autophagy by taking transcriptional effects, like transactivating BH3-only proteins in response to stress; however cytoplasmic P53 might conduct as a major repressor of autophagy by re-localizing to mitochondria, and binding to anti-apoptotic Bcl-2 family members (or activating the proapoptotic Bcl-2 family proteins). Thus, P53 may act to balance autophagy (mitophagy) and apoptosis in regulating cell survival and death in response to stress like stroke injury

Another underlying cause of this duality may be P53, and this is shown in Fig. 2. P53 has overlapping alterations with Bcl-2 family proteins [145], and P53 takes effect mainly in regulating the action of Bcl-2 family proteins in both direct and indirect ways of apoptosis [146]. The expression of BNIP3 can be trans-repressed by P53 directly and therefore to protect cells against apoptotic cell death caused by hypoxia in both vitro and vivo models [147]. Also, P53 plays a dual role in regulating autophagy. Nuclear p53 would induce autophagy by taking transcriptional effects; however cytoplasmic p53 might conduct as a dominant repressor of autophagy [148]. A study conducted in our laboratory(not published) found that, after intracerebral hemorrhage injury, inhibited P53 by electroacupuncture (GV20-GB7) treatment enhanced mitophagy by upregulating Bnip3, and negatively regulated mitochondrial apoptosis. Moreover, immunohistochemistry has shown P53 was mainly expressed in the cytoplasm. These results suggested that, the potential role of P53 working at the balance between the mitophagy and apoptosis, and probably leading to the duality of mitophagy in the pathology.

During, or as a result of stroke, pro-survival and pro-death events are initiated concomitantly in the focal area. The disturbances to the pathways that regulate programmed cell death may determine the tendency of mitophagy towards the pro-survival or pro-death in stroke. In stroke-related research, mitophagy is most often thought of as a cell survival mechanism, since mitophagy removing dysfunctional mitochondria is thought to have contributed to organelle integrity and cell energy maintenance, which is crucial to keeping cellular homeostasis. In a cerebral ischemia rat model, rapamycin-enhanced mitophagy, by facilitating the recruitment of p62 to the damaged mitochondria, which improved mitochondrial function compared with control group, showed improvement of mitochondrial function by decreased malondialdehyde(MDA) and recovered ATP and mitochondrial membrane potential levels. However, these protective effects of rapamycin were reversed by 3MA treatment [149]. Similarly, augmented mitophagy in acute cerebral ischemia (ACI) injury, leading to the results of improved neurological function and reduced the infarct volume and necrosis, the protective effect was by preventing the disruption of the mitochondrial structure and maintaining the MMP. These findings argue for the protective role of mitophagy in stroke [150]. However, a contradictory study has been reported, in which carnosine treatment attenuated p-Drp1 and Parkin and inhibited the release of AIF and cytochrome C at the same time, which indicates that decreasing mitophagy served as mitochondrial protection role since cytochrome C and AIF are released from damaged mitochondria [90].

The reperfusion phase in stroke was suspected to be the dividing crest of the role of autophagy to be neuron protective or destructive during stroke. A study by Zhang & Yan [151], opposite to the condition in the permanent cerebral ischemia, 3-MA inhibited autophagy attenuated rather than elevated ischemia-reperfusion (I-R)-induced neuron injury, and their work also concluded the protective role of autophagy may be achieved by inhibiting mitophagy and decreasing downstream of apoptosis. In the mechanism of autophagy, sublethal ischemic preconditioning (with five minute duration, three episodes, 15 min intervals) activated autophagy showing a protective role against cerebral I-R injury by reducing cleaved caspase-3 expression and decreasing infarct volume, thus improving neurobehavior [152]. A similar result from another study has shown that both electro-acupuncture (EA) treatment preconditioning and postconditioning of spinal cord I-R injury could suppress apoptosis and inhibit neuroinflammation [153]. In light of these findings, the role of pro-conditioning and post-conditioning should be tested and verified, to investigate whether they affect mitophagy in the same way during stroke.

Besides the objective factors and hypothesis described above, the controversial results described in this paper may also be due to the different model types, the time elapsed between stroke induction and observation of mitophagy, different means of intervention or even differences between experimental environments. It is also generally held that the degree of mitochondrial autophagy is the key factor in the role it may play during stroke, and that it can be beneficial to neuronal survival when it is at physiological levels, but could be deleterious when it attains excessive or inadequate levels. Thus, disagreements over the role of mitophagy in stroke should be continuously scrutinized.

## Conclusions

In this review, we provide a comprehensive description of the involvement of mitochondrial autophagy in stroke. At a basic level, mitophagy may responsible for mitochondrial turnover; it may function to clear damaged mitochondria in response to various pathological stresses including stroke [20, 154]. So far, the pathological mitochondrial changes of have been extensively researched in ischemic stroke, but little studied in ICH. A range of research indicates that mitochondrial dysfunction in ischemic stroke might be induced by occlusion of a cerebral artery, or caused by persistent decreased mitochondrial energy metabolism [155, 156]. However, as we have seen, the mechanisms involved, the extent of lesions and pathophysiology of ICH are very different. Accordingly, the research findings of cerebral ischemia may not be applied to haemorrhage stroke. For instance, the oxygen extraction fraction (OEF), which indicates that the state is caused by a primary reduction in brain metabolism, is reduced by mitochondrial dysfunction, rather than ischemia [13].

The advancement of achieving control of mitophagy may result in a shift in stroke care, moving focus to the pre-diagnosis and prognosis of the stroke (in both of ischemic and haemorrhage stroke events). However, understanding of mitophagy mechanism underlying stroke has been limited by preclinical animal studies, and it is imperative that we find out how the mechanism of mitophagy takes place in the human body. The failure to apply neuroprotectant clinical trials to humans may be due to the complexity of stroke pathophysiology, and this should act as a clarion call: we need to consider the comprehensive mechanisms of mitophagy and even the mitophagy-connected crosstalk mechanisms. Of course, there might be other receptors of mitophagy which have not been identified yet, which may also correspond to the pathology of stroke. Broader selective reagents and therapeutic targets for manipulating the mitophagy are needed, and further elucidation of mitophagy and its crosstalk mechanism under stroke conditions is required. Time will tell whether the preclinical research of mitophagy mechanisms in stroke can be used to inform clinical trials. However, it is indisputable that manipulating mitophagy to maintain the integrity and homeostasis of mitochondria is a promising future therapeutic target as scientists seek to preserve neuron viability and to prevent the development of stroke.

## Abbreviations

ACI: Acute cerebral ischemia
ALC: Acetyl-L-carnitine
ASX: Astaxanthin
ATP: Adenosine triphosphate
AV: Autophagic vesicles
BBB: Blood–brain barrier
CCCP: Carbonyl cyanide m-chlorophenylhydrazone
CsA: Cyclosporine A
Drp-1: Dynamin-related protein 1
HEt: Hydroethidine
ICH: Intracranial hemorrhage/ Intracerebral hemorrhage
IPre: Ischemic preconditioning
IR: Ischemia-reperfusion
IV: Intravenous
MCAO: Middle cerebral artery occlusion
MDA: Malondialdehyde
MOMP: Mitochondrial outer membrane permeabilization
MPT: Mitochondrial permeability transition
MPTP: Mitochondrial permeability transition pore
OGD: Oxygen-glucose deprivation
ROS: Reactive oxygen species
SOD: Superoxide dismutase
SOD2: Manganese SOD
tMAO: Transient middle cerebral artery occlusion
tPA: Tissue plasminogen activator
UPS: Ubiquitin-proteasome system
